# Improving Prognostic Stratification in Gastric Cancer: The Role of Lymph Node Staging Systems

**DOI:** 10.3390/medicina62010085

**Published:** 2025-12-31

**Authors:** Tudor Razvan Grigorie, Cosmin Verdea, Teodora Delia Chiriac, Iulia Magdalena Gramaticu, Andreea Iliesiu, George Andrei Popescu, Mihai Popescu, Sorin Tiberiu Alexandrescu

**Affiliations:** 1 Carol Davila University of Medicine and Pharmacy, Dionisie Lupu No. 37, Sector 2, 020021 Bucharest, Romania; andreea.iliesiu@umfcd.ro (A.I.); mihai.popescu@umfcd.ro (M.P.); sorin.alexandrescu@umfcd.ro (S.T.A.); 2 Department of Hepato-Bilio-Pancreatic Surgery, Emergency University Hospital Bucharest, Splaiul Independentei 169, Sector 5, 050098 Bucharest, Romania; verdea.cosmin@gmail.com (C.V.); teodora-delia.chiriac@drd.umfcd.ro (T.D.C.); 3 Department of Oncology, Fundeni Clinical Institute, Sector 2, 022328 Bucharest, Romania; iuliagramaticu@yahoo.com; 4 Department of Pathology, Emergency University Hospital Bucharest, Splaiul Independentei 169, Sector 5, 050098 Bucharest, Romania; 5 Department of Anaesthesia and Intensive Care, Emergency University Hospital Bucharest, Splaiul Independentei 169, Sector 5, 050098 Bucharest, Romania

**Keywords:** gastric cancer, lymph node ratio, log odds of positive lymph nodes, curative-intent gastric surgery, D2 lymphadenectomy, prognostic stratification

## Abstract

*Background and Objectives*: The tumor-node-metastasis (TNM) classification system is the standard for staging gastric cancer and predicting survival. However, its accuracy can be compromised by insufficient lymph node (LN) dissection during surgery or inadequate pathologic examination. Alternative staging systems, such as the lymph node ratio (LNR) and log odds of positive lymph nodes (LODDS), may provide better prognostic value when LN examination is suboptimal. Because the current N staging system was not able to accurately stratify patients relative to their survival outcomes in our series, this study assessed the prognostic impact of LNR and LODDS on overall survival (OS) of patients who underwent radical gastrectomy for cancer. *Materials and Methods*: Between March 2005 and June 2025, the authors performed gastrectomy for gastric carcinoma in 114 patients. Out of these patients, 39 were excluded (19 had stage IV, while 20 underwent palliative gastrectomy with D1 lymphadenectomy). Thus, the study cohort included 75 patients who underwent curative gastrectomy, with 4 (5.3%) of them dying postoperatively. Potential prognostic factors associated with OS (including age, sex, tumor location, T stage, N stage, TNM stage, LNR, and LODDS) were evaluated by univariate and multivariate analysis. Because the recurrence data were missing in 41 patients, the disease-free survival (DFS) analysis would not be meaningful. *Results:* The OS analysis was based on the 71 patients surviving postoperatively. Because successive N stage groups could not accurately stratify patients according to their OS, we used X-tile software version 3.6.1 to identify two cut-offs (both for LNR and LODDS) that were able to stratify patients in three subgroups with significantly distinct survival outcomes. Multivariate analysis found that both LODDS and LNR systems were independent prognostic factors for OS. *Conclusions:* LNR and LODDS provide more detailed insights into lymph node status and have demonstrated potential for enhancing prognostic accuracy compared to N staging, even in patients who underwent curative gastrectomy with D2 lymphadenectomy. Although LNR and LODDS are usually useful in patients who underwent suboptimal lymphadenectomy, the current study demonstrated that these systems could improve prognostic stratification even in patients with more than 15 retrieved LNs. However, due to the small sample size, the current observations and proposed cut-offs of LNR and LODDS have to be validated in larger studies including such patients.

## 1. Introduction

Gastric cancer is a major global health issue, being the fifth-most prevalent cancer worldwide and the fifth leading cause of cancer-related deaths [1,2]. Its incidence varies significantly, being the highest in East Asian countries and in many regions of Central and Eastern Europe. Although cancer rates have steadily declined over the last half-century, mainly for noncardia cancers due to H. Pylori eradication and better food preservation, recent studies have shown rising trends in younger populations, with autoimmune gastritis and gastric microbiome dysbiosis potentially driving tumors near the esophagogastric junction [3,4,5,6,7].

Surgery is the primary treatmesoftnt for early-stage cancers, whereas other therapies, such as chemotherapy, may be administered before and/or after surgery to reduce tumor size or prevent recurrence. Surgical removal of all or part of the stomach remains the cornerstone treatment for gastric cancer. The type of gastrectomy (total, proximal, or distal) depends on the tumor’s location and extent, stage of cancer, and patient factors, with the aim of removing the tumor with clear margins (R0 resection) and preserving healthy tissue and function [8]. Lymph node dissection plays a crucial role in the staging and surgical management of patients with gastric cancer. Therefore, R0 radical gastrectomy with D2 lymphadenectomy is mandatory to achieve radicality in patients with gastric cancer. Although few authors suggested the performance of D2-plus or D3 lymphadenectomy in selected patients, [9,10] none of the current guidelines recommend such an extensive lymph node dissection [11,12,13,14].

The accuracy of staging and prognosis in gastric cancer can be significantly compromised when an insufficient number of lymph nodes (LNs) is examined. Numerous studies have suggested that a minimum of 15 LNs should be retrieved to ensure optimal staging, with over 25 nodes recommended for accurate classification of N stage [11,15,16]. Failure to meet these requirements may result in a phenomenon known as “stage migration” or the Will Rogers phenomenon [17]. However, few authors suggested that, even in patients who underwent radical gastrectomy with D2 lymphadenectomy, lymph node ratio (LNR) and log odds of positive lymph nodes (LODDS) could further refine the prognosis of such patients [15,18,19].

These methods have been proposed in addition to the commonly used staging systems for gastric adenocarcinoma, such as those by the American Joint Committee on Cancer (AJCC) and the Union for International Cancer Control (UICC) [20]. These approaches aim to provide more reliable prognostic information even in cases where the minimum recommended number of LNs was retrieved.

This study aimed to identify the key prognostic factors based on the number of retrieved and positive LNs affecting overall survival (OS) in a cohort of patients who underwent radical surgery for gastric cancer. Thus, we analyzed various clinical and pathological variables using univariate and multivariate statistical methods to assess the prognostic accuracy of pN stage, LNR, and LODDS.

## 2. Materials and Methods

### 2.1. Patients and Data Collection

Patients who underwent surgical intervention for gastric adenocarcinoma between March 2005 and July 2025, with either curative or palliative gastric resection, were retrospectively assessed. It is important to note that a single surgical team conducted operations on these patients from March 2005 to November 2023, at the Fundeni Clinical Institute, and from November 2023 to the conclusion of the study at the Emergency University Hospital Bucharest.

From this cohort, we excluded patients who underwent palliative surgery in the context of metastatic disease and those who underwent only D1 lymphadenectomy because of comorbid conditions. Inclusion criteria were as follows: histologically confirmed gastric adenocarcinoma, non-metastatic gastric carcinoma, and D2 lymphadenectomy. Patients with stage IV gastric adenocarcinoma and those who died during hospitalization for gastric resection or within the first 30 days postoperatively were also excluded from OS analysis.

This retrospective study was approved by the Ethics Committee of Fundeni Clinical Institute, Bucharest, under the number 15384/14.04.2025, and by the Ethics Committee of Emergency University Hospital Bucharest under number 12872/18.02.2025.

### 2.2. Surgical and Oncologic Management

All patients with gastric cancer operated on by our team were evaluated by a multidisciplinary team. The diagnosis was established preoperatively by upper endoscopy and biopsy in all patients. Contrast-enhanced CT of the thorax, abdomen, and pelvis was performed for staging purposes. For TNM staging, we used the UICC 8th edition and performed appropriate conversion in patients whose tumor stage presented with older TNM editions [20]. Especially since 2020, all patients with locally advanced gastric cancer and without primary tumor complications (e.g., digestive stenosis or severe anemia due to hemorrhagic gastric cancer), have benefited from neoadjuvant chemotherapy according to current guidelines [11,13,14,21,22,23]. The most commonly used neoadjuvant chemotherapy regimen was FLOT (docetaxel, oxaliplatin, leucovorin, and 5-fluorouracil).

Staging has been performed according to the 8th Edition of the AJCC staging system.

Follow-up recommendations consist of the evaluation of CEA and CA 19-9 levels, as well as CT scan of thorax, abdomen, and pelvis every 3 months during the first 2 years after operation and every 6 months for the next 3 years. Upper GI endoscopy is recommended at 1 year postoperatively and every 2 years during the next 4 years.

Unfortunately, 41 (57.7%) patients were lost to follow-up, and the only available data regarding their survival outcome were the date of their death or their alive status at 1 July 2025. By this reason, a meaningful analysis of the disease-free survival (DFS) of the patients included in this study cannot be performed.

### 2.3. Statistical Analysis

Upon completion of the database search, the analysis was conducted using IBM SPSS Statistics (Version 27). Continuous variables were reported as mean (±standard deviation) or median [IQR 25–QR 75]. Categorical variables have been presented as number and percentage. The survival outcome of the study was overall survival (OS), defined as the interval between radical gastric resection and either the date of the patient’s death or the study’s conclusion (1 July 2025) for the patients who were alive at that moment. X-tile software version 3.6.1 was used to establish the cut-off values for both LNR and LODDS which could categorize the patients into three subgroups with significantly distinct prognoses. The cut-off values of LNR and LODDS were identified as the values associated with the maximum chi square (and the minimum *p* value).

OS curves were generated using the Kaplan–Meier method. In univariate analysis, the log-rank test was used to compare the OS rates between the groups. All variables with a *p* value < 0.05 at univariate analysis were introduced in multivariate analysis. A backward stepwise Cox proportional hazards regression model was used to identify the independent prognostic factors associated with OS. Hazard ratios (HR) and 95% confidence intervals (CI) were calculated. Statistical significance was set at *p* < 0.05.

## 3. Results

We retrospectively evaluated all the 114 patients who underwent surgery for gastric cancer between March 2005 and July 2025.

**Figure 1:**
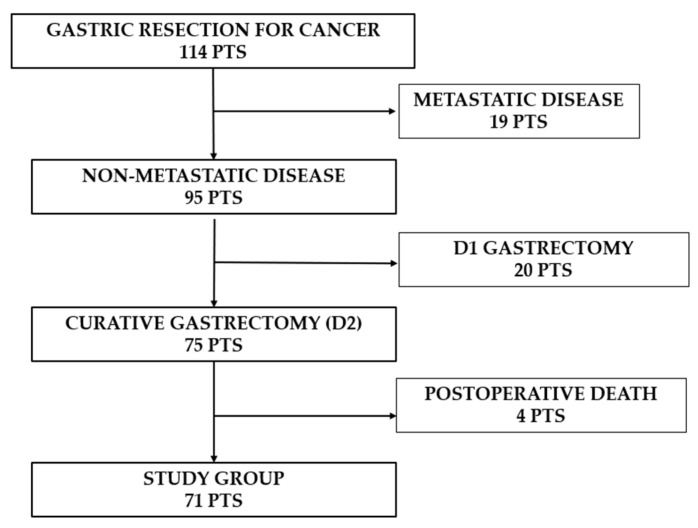
Flowchart showing the number of patients enrolled in the study group.

Out of 114 patients, 39 (34.2%) underwent palliative gastrectomy (19 metastatic patients, 20 underwent only D1 lymphadenectomy), while 75 (65.8%) had radical gastric resection (including D2 lymph node dissection) (Figure 1).

Because the survival outcomes after gastrectomy are significantly poorer in the context of metastatic disease or D1 lymphadenectomy, we further evaluated the short-term postoperative outcomes only for non-metastatic patients undergoing D2 lymphadenectomy (75 patients).

### 3.1. Clinico-Pathologic Characteristics of the Entire Group

The average age of the patients was 62.6 years, with a standard deviation of 9.5. The patient group was categorized into those aged 65 or younger (69.2%) and those older than 65 (30.8%), highlighting the predominance of younger patients in this cohort (Table 1).

The study population comprised a higher proportion of males (62.7%) compared to females (37.3%). The antrum was identified as the most common site for tumor occurrence (53.3%), followed by the body of the stomach (41.3%) and a smaller proportion of patients had the primary tumor located at the gastroesophageal junction (GEJ) (5.4%). The cohort was nearly equally divided between those undergoing total gastrectomy (50.6%) and those undergoing subtotal gastrectomy (49.4%).

The majority of patients (76%) did not receive neoadjuvant chemotherapy, whereas 24% (18 patients) did, suggesting that most patients proceeded directly to surgery without prior chemotherapy.

**Figure 2:**
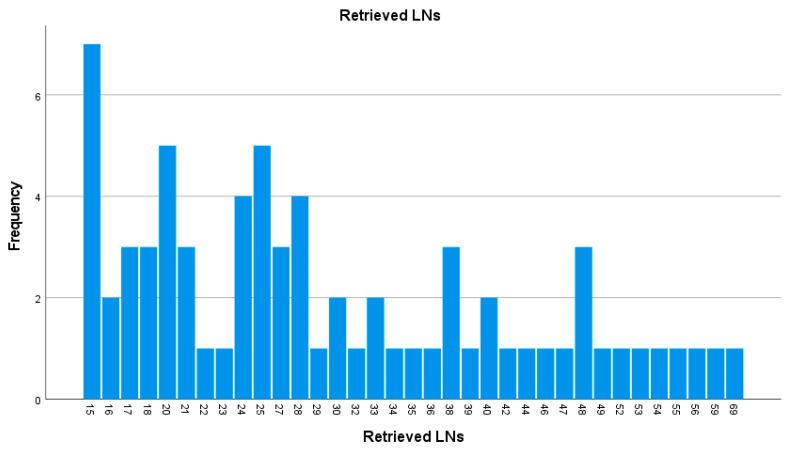
Histogram displaying the distribution of total nodes examined per patient (LNs—lymph nodes).

The median number of harvested LNs per patient was 26 [IQR 20–39]. The distribution of evaluated LNs is presented in Figure 2.

A significant proportion of patients had no lymph node involvement N0 (38.6%), yet 61.4% had positive nodes—N1 (18.6%), N2 (22.7%), and N3 (20.1%).

Patients were distributed across various stages, with Stage IIA being the most common (28%), followed by Stage IIIA (24%), indicating that the study population largely consisted of patients with advanced-stage, non-metastatic cancer (Table 1).

### 3.2. Short-Term Outcomes

Postoperative complications that occurred during hospitalization or 30 days after the surgical procedure were assessed using the Dindo Clavien grading system (Table 1) [24,25,26]. While 47 (62.6%) patients had an uneventful postoperative course, 21 (28%) patients developed minor postoperative complications and 7 (9.3%) had major complications eventually leading to the death of 4 (5.3%) patients.

### 3.3. Setting of LNR and LODDS Cut-Off Values

Because 4 patients died postoperatively due to other causes than malignancy’s progression, the survival analysis was based on 71 patients who survived after operation.

**Figure 3:**
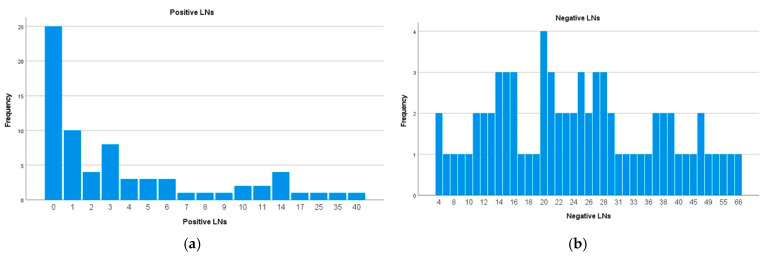
(**a**) Number of positive LNs per patient. (**b**) Number of negative LNs per patient.

The median number of positive LNs per patients was 2 [IQR 0–6], and the median number of negative LNs was 24 [IQR 15–35] (Figure 3).

LNR represents the proportion of the positive LNs from the entire number of examined LNs. LODDS was calculated using the following formula: LODDS = log ([NPLN +  0.5]/[NDLN-NPLN +  0.5]), where NPLN is the number of positive LNs and NDLN is the number of dissected LNs. X-Tile software (version 3.6.1; Yale University School of Medicine, New Haven, CT, USA) was used to identify the optimal LODDS and LNR cut-off values able to stratify patients into three groups with significantly different OS rates. By using X-tile software, we determined the two cut-offs associated with the maximal survival difference (or the highest log-rank χ^2^ value) among the three groups [27].

**Figure 4:**
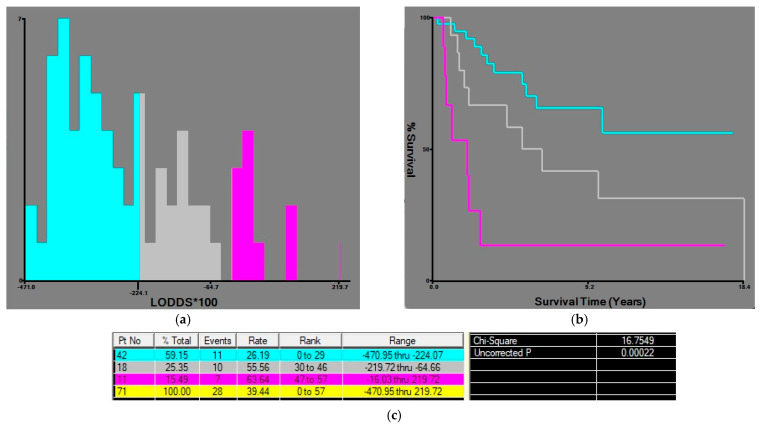
X-tile plots of the LODDS*100 (log odds of positive lymph nodes multiplied by 100) and corresponding OS curves, in patients with radical gastrectomy for gastric adenocarcinoma. (**a**) Histogram showing LODDS: the values of −2.2 and −0.6 divided the patients into three subgroups. (**b**) Comparative survival curves of the three subgroups. (**c**) OS rates were associated with the highest chi square and the lowest *p* value. Blue bars/curve—LODDS0 group, gray bars/curve—LODDS1 group, purple bars/curve— LODDS2 group.

For LODDS, the two cut-off points were established at −2.2 and −0.6 and the patients were divided into three groups according to the LODDS value: LODDS 0 ≤ −2.2, LODDS 1 > −2.2, ≤ −0.6, and LODDS 2 > −0.6 (Figure 4).

**Figure 5:**
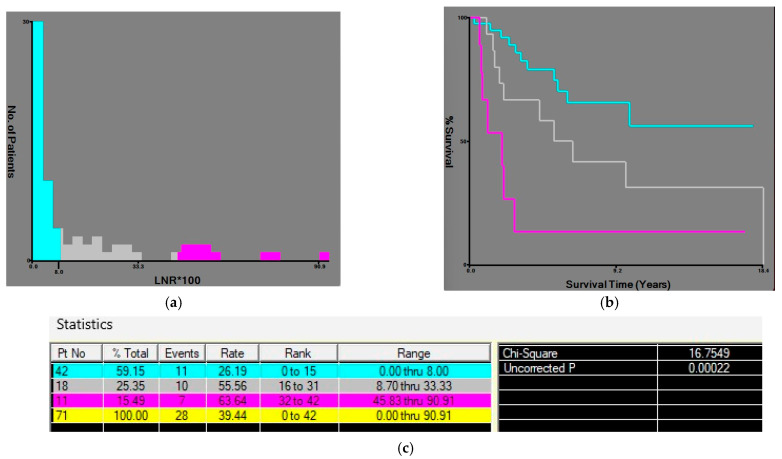
X-tile plots of the LNR*100 (lymph node ratio multiplied by 100) and corresponding OS curves, in patients with radical gastrectomy for gastric adenocarcinoma. (**a**) Histogram showing LNR: the value of 0.08 and 0.33 divided the patients into three subgroups. (**b**) Comparative survival curves of the three subgroups. (**c**) OS rates were associated with the highest chi square and the lowest *p* value. Blue bars/curve—LNR0 group, gray bars/curve—LNR1 group, purple bars/curve—LNR2.

The same method was used to find cut-off values for LNR, also establishing two values at 0.08 and 0.33; patients were divided according to the LNR value into three groups: LNR0 ≤ 0.08, LNR1 > 0.08, ≤ 0.33 and LNR2 > 0.33 (Figure 5).

### 3.4. Long-Term Outcomes (OS)

For the study group of 71 patients, the median OS was 117 months, with 1-, 3-, 5- and 10-year OS rates of 92.3%, 71.2%, 65.1%, and 43.6%, respectively.

To evaluate the factors significantly associated with OS, we initially performed a univariate analysis and, subsequently, multivariate analysis.

The variables included in univariate analysis were demographic data (age and sex), treatment factors (neoadjuvant chemotherapy, type of gastrectomy), tumor characteristics (location, grading, T-, N-, and TNM stage), postoperative complications, LNR, and LODDS.

Several factors did not have a statistically significant association with OS in univariate analysis (Table 2), while poor differentiation, higher N stage, higher LNR and LODDS were all significantly associated with poorer OS (Table 3).

**Figure 6:**
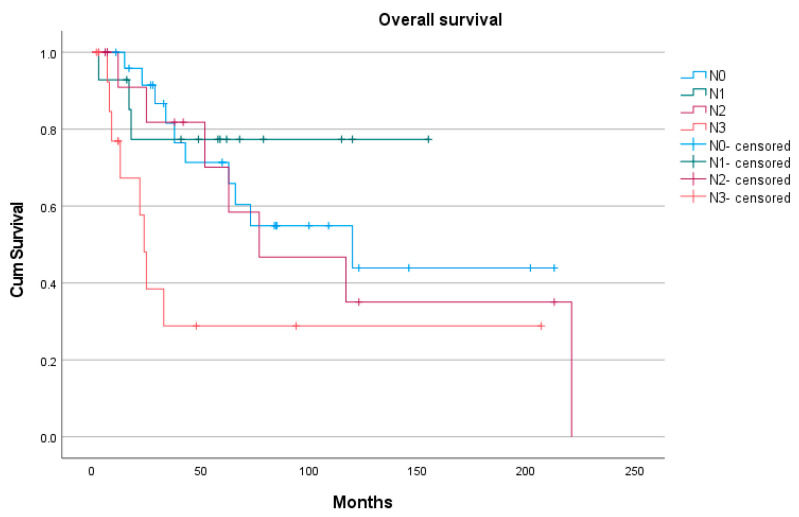
Comparative OS rates according to the N stage.

However, when consecutive N stages have been compared one by one, the difference in OS was not statistically significant. Thus, the median OS for N0 vs. N1 was 120 months vs. NR (*p* = 0.443). Between the N1 and N2 stages, the difference in median OS was also not statistically significant (NR vs. 77 mo.; *p* = 0.372). Furthermore, the difference in OS was not significant between the N2 and N3 stages (77 vs. 24 mo.; *p* = 0.103). Thus, the N stage could not adequately stratify patients relative to their OS (Figure 6).

By this reason, we used other staging systems based on the number of evaluated LNs and positive LNs, such as LNR and LODDS, to adequately stratify the patients relative to their overall survival outcome.

In multivariate analysis, tumor grading was not an independent factor associated with prognosis (*p* = 0.474). Regarding the N stage, only the N3 stage was an independent factor associated with significantly poorer OS (HR = 54.502, 95% CI: 2.188–1357.612, *p* = 0.015), while N1 and N2 were not independent risk factors (for N1-stage: HR = 1.361, 95% CI: 0.163–11.394; for N2-stage: HR = 11.465, 95% CI: 0.964–136.329; *p* > 0.05 for both). Both LNR and LODDS were independent factors associated with prognosis, and the two cut-offs determined by X-tile software were able to stratify patients into three categories, each of them being independently associated with a significantly distinct prognostic (LNR/LODDS1: HR = 52.450, 95% CI: 4.354–631.774, *p* = 0.002; LNR/LODDS2: HR = 260.715, 95% CI: 9.834–6912.215, *p* < 0.001) (Table 3).

## 4. Discussion

Gastric cancer remains one of the leading causes of cancer worldwide, ranking among the top five leading causes of cancer-related mortality [1,28]. Without surgery, the overall survival rate at 5 years is dismal (less than 5%), while in patients, radically resected 5 year OS rates increase, according to stage of the disease, from 36–50% in stage III to 70% in stage II patients and over 90% in early gastric cancer [29,30,31].

In our study group, the majority of patients had locally advanced disease with 5-year OS rates of 77.1%, 72%, and 51.8% in stages I, II, and III, respectively, similar to survival rates reported in previously mentioned studies [29,30,31,32].

Traditional staging systems, such as the UICC/AJCC systems, do not always accurately estimate OS. Numerous studies have suggested that the use of alternative staging systems, either alongside or instead of the classical TNM staging system, can improve the prognostic estimation of survival. The traditional TNM staging system shows limited prognostic efficacy when data collection is inadequate, adherence to guidelines is lacking, in instances of early gastric cancer in Asian countries [33,34], as well as in situations of stage migration following neoadjuvant chemotherapy, or when fewer than 15 LNs are retrieved or examined after radical D2 gastrectomy.

Lymph node dissection is crucial in radical gastrectomy, especially in advanced stages, because it allows for more accurate staging and potentially curative removal of the disease. However, studies conducted in the USA from 1998 to 2005 indicated that only 29% of patients had more than 15 nodes assessed [15,35,36,37,38]. For such instances, when postoperative LN assessment was not adequate, alternative staging systems such as LNR and/or LODDS were released.

LNR was initially utilized in breast cancer studies, whereas the LODDS was first introduced for breast cancer and subsequently validated in other cancers. Both are relatively new prognostic systems that have been applied to various cancers, including gastric, pancreatic, and colorectal cancer. Consequently, the LNR and LODDS staging systems have emerged as more significant prognostic factors than the pN (pathological node status) or TNM staging systems, especially in cases with inadequate lymph node dissection [39,40].

In our study, although all patients had at least 15 LNs evaluated, the N stage was not able to adequately stratify patients relative to the OS. This observation is similar to those reported by other authors who recommended the assessment of a minimum of 25 LNs in curative-intent operations performed for gastric cancer, to achieve an adequate prognostic stratification based on TNM staging [11,15,16]. Because 40.8% (29/71) of the patients included in the current study had between 15 and 24 evaluated LNs, and the N stage cannot adequately stratify the patients, we decided to explore the prognostic value of LNR and LODDS in our group of patients who underwent radical gastrectomy with D2 lymphadenectomy.

Our work showed that LNR and LODDS were independent prognostic factors of survival according to multivariate analysis, similar to the findings of Jian-Hui et al. [18].

Other studies have also shown an important relationship between the LNR and LODDS [15,19,41]. They demonstrated, as in our study, that both LNR and LODDS are superior to the pN classification in discriminating the different prognoses of patients, but not in a linear manner, although they are strongly correlated [42]. In these studies, the LODDS system had the potential to discriminate between patients with the same LNR and remained the most appropriate staging system [15,18,41].

In our study, possibly due to the limited sample size, the LODDS did not demonstrate superiority over the LNR, with both staging systems exhibiting equivalent prognostic values. With a larger, more diverse dataset, the two markers might have shown distinct, independent prognostic value, as reported in other studies [15,19,41]. In the context of the small sample size, the modality to determine the cut-off points with X-tile might explain the fact that software found the cut-off values for both LNR and LODDS at levels that divided the entire group into three identical subgroups for corresponding LODDS and LNR (the same patients were included in LNR0 and LODDS0, LNR1 and LODDS1, LNR2 and LODDS2). This is the explanation for the perfect collinearity of LNR and LODDS in this series, and it represents a significant limitation of the current study.

However, there are numerous modalities for calculating cut-off points for stratifying patients according to lymph node status using the percentile method, ROC (Receiver Operating Characteristic) curve analysis, AIC (Akaike Information Criterion), log-rank test and X-tile software [19,42,43,44,45,46]. Each of these methods has strengths and limitations, and the choice of method can significantly affect the resulting stratification. While other methods like ROC curve analysis are useful for assessing the trade-off between sensitivity and specificity, and AIC is good for model quality assessment, X-tile’s direct focus on identifying prognostically significant survival differences made it a suitable tool for achieving this study’s primary objective of improving prognostic stratification. The X-tile method was used in our study alongside the one conducted by Zeng et al. [46] who categorized patients into four subgroups (whereas our study identified three subgroups owing to the smaller sample size).

Regardless of the chosen method, the ultimate objective remains consistent: to create patient subgroups that demonstrate distinct prognostic outcomes, thereby enhancing their ability to predict survival and inform treatment decisions and follow-up regimen.

The frequently used cut-off of LNR set at 0 does not bring any prognostic benefit for N0 patients, and this is the explanation of the fact that the lower cut-off of LNR in our study was identified by X-tile software at 0.08. Similarly, Çapkinoğlu et al. used a single cut-off of 0.09 which divided 193 patients (median of 24 harvested LNs) into two groups with distinct survival outcomes [47]. Our higher LNR cut-off of 0.33 identified with X-tile software was similar to those reported by other studies [48,49,50]. For example, Wang et al. divided 18,043 patients included in the SEER database into four subgroups with significantly different prognosis using the cut-offs of 0.07, 0.3, and 0.7 [51]. In our study, the cut-offs were similar to their lower cut-offs, but because our series has a low sample size, the introduction of a third cut-off would generate four subgroups with a very small number of patients. Regarding the usefulness of LODDS in stratifying patients with gastric cancer, most studies revealed that, in patients with more than 15–20 LNs retrieved, LODDS lost its prognostic significance [15,44]. Thus, Cao et al. found in 877 patients who underwent D2 gastrectomy that the C-index of LODDS was significantly lower than those of LNR when more than 15 LNs have been harvested [44], while Sun et al. observed in 1593 patients with more than 15 evaluated LNs that LODDS lost its significance and was substituted by the N stage [15]. Their results may be explained by the cut-offs used, which cannot adequately stratify the patients with more than 15–20 retrieved LNs (Cao used the cut-offs of −0.5, 0, 0.5, while Sun used the cut-offs of −1.5, −1, −0.5 and 0). Due to the mathematical formula used to calculate LODDS, when more than 15 LNs are evaluated, most patients will be included in the group with very low LODDS value (e.g., less than −1.5 or less than −0.5), and a very small number of patients will be included in the other groups. For example, in our series, 50 (70.4%) patients had LODDS lower than −1.5, 6 (8.5%) had LODDS between −1.5 and −1, 4 (5.6%) patients were between −1 and −0.5, 3 (4.2%) patients had LODDs ranging between −0.5 and 0, 5 (7%) patients had LODDS between 0 and 0.5, and 3 (4.2%) patients had LODDS higher than 0.5. It is obvious that a more balanced number of patients in each group should be achieved by moving the cut-offs toward lower levels, explaining why, in our series, X-tile software found lower levels that were able to divide patients in homogeneous prognostic subgroups. Different from our method to determine cut-offs by using X-tile software, which discriminates patients according to their survival expectancy, most of the above-mentioned authors used rather empirical cut-offs, with LODDS classification intervals being determined by comparing OS rates according to LODDS with an initial interval of 0.5 and combining patients with similar prognosis [15,44]. Our cut-offs for LODDS were much more similar to those of Lee et al. who divided 3929 patients (97% of whom had more than 15 evaluated LNs) into five groups using the cut-offs of −4, −2.5, −2, and −0.5 [42]. The cut-offs that we used (−2.2 and −0.6) roughly included in our first subgroup (less than −2.2) their LODDS 1 and LODDS 2, in the second subgroup (−2.2 to −0.6) their LODDS 3 and LODDS 4, and our last subgroup was similar to their LODDS5 group. The similarity of our cut-offs with those of Lee may be explained by the fact that, in their series, almost 97% of patients had more than 15 retrieved LNs, similar to our cohort. Moreover, although they found that LODDS staging facilitates more accurate prognostic stratification, their results revealed that the receiver operating characteristic (ROC)-AUC curves of N-, LNR-, and LODDS staging systems did not differ significantly in terms of predicting survival [42]. This observation corroborated the previously discussed modality of achieving our cut-offs, may explain the collinearity between LNR and LODDS in our series, and might be a characteristic of the cohorts including patients with sufficient harvested LNs.

Thus, the current study revealed that cut-offs of LNR, and especially LODDS, that are able to discriminate patients into distinct prognostic groups with significantly different OS outcomes should be different in patients with more than 15 evaluated LNs than those frequently used for patients with insufficient retrieved LNs.

### 4.1. Limitations

One limitation of our study is the relatively small cohort size which precluded the possibility to separately evaluate the patients who underwent upfront surgery vs. those who received neoadjuvant chemotherapy. The cohort size of 18 patients receiving neoadjuvant therapy before surgery is inadequate for further subdivision. Furthermore, the follow-up would be too short, since all these patients were operated during the last 5 years. In this context, our results rather reflect the discriminative ability of LNR and LODDS in patients with gastric carcinoma who underwent upfront D2 gastrectomy. Stage migration in patients receiving neoadjuvant chemotherapy for gastric cancer presents a significant challenge for accurate prognostication using the current staging systems. The effectiveness of neoadjuvant treatment can lead to tumor downstaging and a reduction in lymph node involvement, potentially altering the patient’s staging classification. This phenomenon underscores the need for a new and more adaptive staging system that accounts for the effects of preoperative therapy. Distinct studies including only patients who underwent neoadjuvant therapy are mandatory to determine whether LNR and LODDS staging systems could be useful in the prognostic stratification of such patients and eventually determine the adequate cut-offs. The usefulness of TNM staging, LNR, and LODDS is even more debatable in patients receiving preoperative immunotherapy and chemotherapy, since almost 20% of them had complete pathologic response [52]. Also, due to the small sample size, the cut-off values of LODDS and LNR found in the current study cannot be validated in an independent cohort of patients.

Another limitation is the retrospective nature of the study. As a consequence, follow-up data regarding the time and type of recurrence were available only for 30 patients (42.3%), thus precluding the possibility to evaluate disease-free survival (DFS) in our cohort.

Due to these important limitations, this study should be considered as a pilot study, with future studies being necessary to validate its results.

### 4.2. Future Directions

Starting from these results, a future larger study involving hundreds of patients with non-metastatic gastric cancer undergoing radical gastrectomy should be performed. Because neoadjuvant therapy is the standard of care in present, the patients should undergo preoperative oncologic therapy. The study protocol should include preoperative TNM staging, surgery should include adequate D2 lymphadenectomy performed by surgeons experienced in gastric cancer operations, pathologic evaluations should follow a dedicated protocol to adequately identify all yielded LNs, and a strict follow-up protocol to determine the time and location of recurrence should be implemented. After a median follow-up of more than 36 months, the OS and DFS results should be evaluated according to the N stage, LNR, and LODDS. The cut-off values of LNR and LODDS should also be determined with X-tile software, and their prognostic accuracy must be evaluated with the ROC curve. Further, these cut-offs should be applied to a distinct series of similar patients, to validate the results achieved in the control group. However, because a randomized controlled trial which demonstrated that preoperative association of immunotherapy to the FLOT regimen significantly improved survival outcomes was published this year [52], it would be better to use this combination therapy preoperatively instead of neoadjuvant chemotherapy alone.

## 5. Conclusions

In patients who underwent radical D2 gastrectomy, LNR and LODDS were able to better stratify patients relative to their OS than the current TNM staging system. Although previous studies suggested that LNR and LODDS are useful, mainly in case of insufficiently evaluated LNs; the current results emphasize that these staging systems could also be applied in patients with an adequate number of retrieved LNs. However, this should be considered as a pilot study due to the small sample size, requiring further validation in larger studies with more datapoints, especially in terms of the accuracy of these staging systems in patients who underwent preoperative oncologic therapy.

## Figures and Tables

**Table 1:** Clinico-pathological characteristics of all patients.

Characteristics	No. of Patients (%)
Age (Mean ± SD)	62.6 (±9.5)
≤65	45 (69.2%)
>65	30 (30.8%)
Sex	
Male	47 (62.7%)
Female	28 (37.3%)
Location	
GEJ	4 (5.4%)
Body	31 (41.3%)
Antrum	40 (53.3%)
Neoadjuvant chemotherapy	
Yes	18 (24%)
No	57 (76%)
Gastrectomy	
Total	38 (50.6%)
Subtotal	37 (49.4%)
TNM stage	
IA	8 (10.6%)
IB	5 (6.6%)
II A, B	31 (41.4%-A-28%, B-13.4%)
III A, B, C	31 (41.4%-A-24%, B-12%, C-5.4%)
N stage	
N0	29 (38.6%)
N1	14 (18.6%)
N2	17 (22.7%)
N3	15 (20.1%)
Tumor grade	
G1	8 (10.6%)
G2	25 (33.4%)
G3	42 (56%)
LODDS	
LODDS0. ≤ − 2.2	46 (61.4%)
LODDS1. > −2.2, ≤−0.6	18 (24%)
LODDS2. > −0.6	11 (14.6%)
LNR	
LNR0. ≤ 0.08	46 (61.4%)
LNR1. > 0.08 ≤ 0.33	18 (24%)
LNR2. > 0.33	11 (14.6%)
Dindo Clavien	
Grade 0	47 (62.66%)
Grade 1	6 (8%)
Grade 2	15 (20%)
Grade 3a	1 (1.33%%)
Grade 3b	2 (2.66%)
Grade 5	4 (5.33%)

SD—standard deviation; GEJ—gastroesophageal junction; LODDS—log odds of positive lymph nodes; LNR—lymph node ratio.

**Table 2:** Univariate analysis of variables that did not show statistical significance (*p* > 0.05).

Characteristics of the Patients	Univariate Analysis *p* Value
Gender	0.365
Male	
Female	
Age	0.562
≤65	
>65	
Neoadjuvant CHT	0.206
Yes	
No	
Tumor location	0.366
GEJ	
Body	
Antrum	
Gastrectomy	0.972
Total	
Subtotal	
Postoperative	0.908
Complications	
Yes	
No	
UICC	0.148
Stage I	
Stage II	
Stage III	
UICC	0.051
Stage I-II	
Stage III	
Tumor stage	0.246
T1	
T2	
T3	
T4	

CHT—chemotherapy; GEJ—gastroesophageal junction; UICC—Union for International Cancer Control.

**Table 3:** Multivariate analysis of variables with statistical significance in univariate analysis.

Variable	Univariate Analysis *p* Value	Multivariate Analysis		
		HR	95% CI	*p* Value
Tumor grading	0.026			
Grade 1, 2		1		
Grade 3		1.395	0.561–3.465	0.474
N stage	0.029			
N0		1		
N1		1.361	0.163–11.394	0.776
N2		11.465	0.964–136.329	0.053
N3		54.502	2.188–1357.612	***0.015***
LNR	<0.001			
LNR0 ≤ 0.08		1		
LNR1 0.081–0.33		52.450	4.354–631.774	***0.002***
LNR2 > 0.33		260.715	9.834–6912.215	***<0.001***
LODDS	<0.001			
LODDS0 ≤ −2.2		1		
LODDS1 −2.2–−0.6		52.450	4.354–631.774	***0.002***
LODDS2 > −0.6		260.715	9.834–6912.215	***<0.001***

Bold and italic—values with statistical significance; CI—confidence interval.

## Data Availability

The original contributions presented in this study are included in the article. Further inquiries can be directed to the corresponding author(s).

## Abbreviations

The following abbreviations are used in this manuscript:

TNM	Tumor-Node-Metastasis
LN	Lymph Node
LNR	Lymph Node Ratio
LODDS	Log Odds of Positive Lymph Nodes
AJCC	American Joint Committee on Cancer
UICC	Union for International Cancer Control
OS	Overall Survival
FLOT	Fluorouracil, Leucovorin, Oxaliplatin, and T docetaxel
HR	Hazard Ratio
CI	Confidence Intervals
EGJ	Esophagogastric junction
NPLN	Number of Positive Lymph Nodes
NDLN	Number of Dissected Lymph Nodes
pN	Pathological Node Status
ROC	Receiver Operating Characteristic
AIC	Akaike Information Criterion
